# Case Report: Prenatal Diagnosis and Treatment of Fetal Autoimmune-Associated First-Degree Atrioventricular Block: First Report From China

**DOI:** 10.3389/fcvm.2021.683486

**Published:** 2021-06-21

**Authors:** Changqing Tang, Haiyan Yu, Shuran Shao, Yimin Hua, Maoli Chen, Qi Zhu, Yifei Li, Kaiyu Zhou, Chuan Wang

**Affiliations:** ^1^Department of Paediatric Cardiology, West China Second University Hospital, Sichuan University, Chengdu, China; ^2^The Cardiac Development and Early Intervention Unit, West China Institute of Women and Children's Health, West China Second University Hospital, Sichuan University, Chengdu, China; ^3^West China Medical School, Sichuan University, Chengdu, China; ^4^Department of Obstetrics, West China Second University Hospital, Sichuan University, Chengdu, China; ^5^Key Laboratory of Birth Defects and Related Diseases of Women and Children (Sichuan University), Ministry of Education, Chengdu, China; ^6^Key Laboratory of Development and Diseases of Women and Children of Sichuan Province, West China Second University Hospital, Sichuan University, Chengdu, China; ^7^The People's Hospital of Yaan, Yaan, China; ^8^Department of Ultrasonaography, West China Second University Hospital, Sichuan University, Chengdu, China

**Keywords:** fetal atrioventricular block, autoimmune disease, first-degree atrioventricular block, treatment, diagnosis, fetus

## Abstract

**Background:** The rapid progression from fetal first-degree atrioventricular block (AVB) to third-degree AVB had been reported. However, how to define fetal first-degree AVB with proper technique and the necessity of the treatment *in utero* for fetal autoimmune-associated first-degree AVB are still controversial.

**Purpose:** To explore the diagnosis and the effect of treatment for fetal first-degree AVB.

**Cases Presentation:** Four pregnant women with positive autoantibodies anti-SSA/Ro were admitted into our hospital with complaints of rapid prolonged atrioventricular (AV) intervals of their fetuses. Fetal AV intervals were re-measured by tissue Doppler imaging (TDI) from the onset of atrial contraction to ventricular systole (Aa-Sa), which were 170 ms (case 1-twin A), 160 ms (case 1-twin B), 163 ms (case 2) and 172 ms (case 3) and 170 ms (case 4), respectively. The histories of medication usage or infection during gestation were denied. Amniotic fluid genetic screenings and virological tests were negative in all cases. No structural cardiac disorders were found and the cardiovascular profile scores were 10 for each fetus. Oral dexamethasone (initial dose of 4.5 mg daily) and hydroxychloroquine (200 mg bid) plus weekly follow-up surveillance were suggested. The dosage of dexamethasone was adjusted according to the changes of the AV intervals and fetal development of biparietal diameters (BPD) and femur lengths (FL). All fetal AV intervals were controlled well. Maternal and fetal adverse effects were noted as diabetes in 1 mother and growth retardation in all fetuses. All fetuses were delivered *via* cesarean section at 35^+4^, 37, 38, and 37^+1^ gestational weeks, with 10 scores of Apgar score. Postnatally, positive anti-SSA/Ro was found in all neonates. However, there were no clinical or laboratory evidence of neonatal lupus syndrome. No abnormal signs were found on postnatal electrocardiogram and echocardiography for all neonates. With a follow-up of 8–53 months, there was no progression of disease and all infants demonstrated normal physical, mental, and motor development.

**Conclusion:** Prenatal treatment for fetal autoimmune-associated first-degree AVB could be an alternative. Strict surveillance and timely adjustment of the treatment according to the conditions of the mother and the fetus are indicated. Further studies are necessary to prove our concept.

## Introduction

Congenital heart block (CHB) is a rare and life-threatening disorder with an incidence of 1 in 15,000–20,000 live births, of which more than half are associated with maternal autoimmune disease (1). Autoimmune-associated CHB, which often develops during gestational weeks (GWs) 16–24, is attributable to the transplacental transport of maternal autoantibodies, especially anti-Ro/SSA and anti-La/SSB, which are detected in 87% of autoimmune CHB cases (2–4). Although only 1–2% of mothers with anti-Ro/SSA and/or anti-La/SSB autoantibodies give birth to a child suffering from CHB, the risk increases to 17–19% in subsequent pregnancies (5, 6). Additionally, >80% of cases of CHB are diagnosed as third-degree atrioventricular block (AVB) and pacemaker implantation is required in more than 65% of surviving newborns with an overall mortality rate of 4–29% (1, 4).

Although emerging evidence suggests that first-degree, second-degree and/or high-degree AVB may be converted and its progression to third-degree AVB may be prevented if it is diagnosed and treated early, a rapid progression to third-degree AVB has also been reported (5–9). Second-degree/high-degree AVB is often treated, however the most beneficial and proper management strategy for first-degree AVB remains controversial due to unstandardized diagnostic criteria, variable outcomes, and most importantly, limited data. The optimal management of AVB *in utero* is yet to be established.

In this study, we firstly present the experience of successful reversal of first-degree AVB through our management in four cases with positive anti-SSA/Ro antibodies in China, attempted to provide a reference for the clinical management of these patients.

## Ethics Statement

Informed written consent was obtained from the patients (and guardians) for the publication of this case report after the nature of this study had been fully explained to them. The study was approved by the University Ethics Committee on Human Subjects at Sichuan University (2010002).

## Methods

### Subjects

Four pregnant women were referred to our center with complaints of rapid prolonged atrioventricular (AV) intervals of their fetuses.

### Prenatal Assessment

A detailed echocardiographic examination was performed by experienced fetal cardiologists using Vivid-7 (GE medical systems) ultrasound systems with 2.0–4.0 MHz curved-array transducers. Two-dimensional guided tissue Doppler imaging (TDI) was obtained from a cardiac four-chamber view. TDI-derived AV interval could be measured from the atrial contraction (Aa) to isovolumetric contraction (IV) or to ventricular systole (Sa) at the base of the right ventricular wall. However, in clinical work, we found that it is relatively challenging to obtain a clear waveform of isovolumetric contraction and identifying the onset of isovolumetric contraction is difficult and always inaccurate. The reproducibility of Aa-Sa measurement was much better than that of Aa-IV. Therefore, TDI-derived Aa-Sa was applied to determine fetal AV intervals and identify fetal first-degree AVB in our center. Measurements were obtained from five consecutive cardiac cycles and subsequently averaged. Except for the fetal AV interval, we sought evidence on fetal echocardiography of anti-Ro/SSA antibody-mediated cardiac disease, including dilated cardiomyopathy, pericardial effusion, pleural effusions or ascites, and endocardial fibroelastosis (EFE). Dilated cardiomyopathy was defined as ventricular dysfunction and cardiac dilation. EFE was defined as presence of areas of increased, patchy echogenicity in the atria, AV valve apparatus or ventricles.

Our group has previously determined the normal values of TDI-derived Aa-Sa intervals (in Chinese) (10). A total of 111 fetuses from different gestational weeks were prospectively recruited. They were divided into four groups according to the gestational week: 20–24 GWs (*n* = 20), 25–29 GWs (*n* = 37), 30–34 GWs (*n* = 32), and 35–42 GWs (*n* = 22). The corresponding normal values of Aa-Sa described as mean ± standard deviation (SD) in these four groups were 108.80 ± 5.08 ms, 111.74 ± 6.27 ms, 122.02 ± 8.54 ms, and 123.39 ± 5.96 ms, respectively. The highest 2SD above the upper limit of normal for the Aa-Sa interval was 139.10 ms, and the highest 3SD above the upper limit of normal (>99% CI) was 147.64 ms. The definition of abnormal fetal TDI mechanical AV interval was set a priori at 3SD (>150 ms), rather than 2SD, above the normal mean. A more stringent criterion for defining abnormal was used in our group because we envisioned this threshold to represent a clinical trigger for labeling the fetus with a serious disease and potentially treating the maternal-fetal dyad with a drug associated with serious toxicity (maternal dexamethasone). Additionally, a previous study (11) has found that the AV time measurement by Aa-Sa was longer than that of fetal PR interval since the ventricular pre-ejection period (time delay from Q wave to ventricular ejection) is longer than the atrial pre-ejection period (time delay from P wave to atrial ejection). Moreover, the study conducted by Sonesson et al. (12) had found that fetal AV intervals in the range of 135–140 ms spontaneously reverted and were not associated with clinical pathology.

### Prenatal Treatment

On the diagnosis of fetal autoimmune-associated first degree AVB, particularly those with EFE, dexamethasone (4.5 mg per day as the initial dosage) and hydroxychloroquine (HCQ, 200 mg two times per day) were administrated to the mother of the affected fetus until delivery. After initiation of the treatment, weekly follow-up of fetal AV intervals, cardiac function, fetal development as well as monitoring of maternal side effects of dexamethasone and HCQ were performed. If the fetal AV interval declined to normal range, the dosage of dexamethasone was decreased to 3.75 mg per day and subsequently be reduced once every 2–4 weeks by 0.75 mg each time, and be maintained at 1–2 mg until delivery. However, obvious signs of fetal growth restriction and decreasing volume of amniotic fluid were considered indications to decrease the dosage at a faster rate.

### Postnatal Assessment

Comprehensive postnatal examination of all affected newborns was performed postnatally. Blood routine test, liver function, titers of anti-Ro/SSA and anti-La/SSB antibodies, ECG as well as the echocardiography were scheduled at birth. Thereafter, monthly follow-up during the first year of life and twice a year afterwards were suggested.

## Results

### Maternal Features

The clinical characteristics of both the mothers and children were described in Table 1. The ages of four mothers were 32 (case 1), 43 (case 2), 25(case 3), 27 (case 4) years old, respectively. Positive anti-SSA/Ro-52 autoantibody was confirmed in all mothers by enzyme-linked immunosorbent assay (ELISA). Except for anti-SSA/Ro-52, antihistone antibody (AHA) and antinuclear antibodies (ANA) were positive in case 2 as well as ANA and proliferating cell nuclear antigen (PCNA) in case 4. The first woman was diagnosed as connective tissue disease (CTD) but did not present with any clinical manifestation, while she had difficulty becoming pregnant and two failed cycles of *in vitro* fertilization. The second mother suffered from systemic lupus erythematosus (SLE) for over 10 years and was treated with oral prednisone (10 mg qd), with two failed gestations (G2P0). The third woman was asymptomatic all the time without the history of pregnancy. The case 4 was previously diagnosed as Sjogren's syndrome and had given birth to a healthy child (G2P1).

**Table 1:** Clinical characteristics of five fetuses with fetal autoimmune-associated first-degree AVB in our center.

**Cases**	**Age (years)**		**G (n.) P (n.)**		**Autoimmune diseases**		**Comorbid disease**		**Positive autoantibodies**	
**Mothers**										
Case 1-twin A		32		G1P2		CTD		None		Anti-SSA/Ro-52
Case 1-twin B										
Case 2		43		G3P1		SLE		Gestational diabetes		Anti-SSA/Ro-52, AHA, ANA
Case 3		25		G1P1		Asymptomatic		None		Anti-SSA/Ro-52
Case 4		27		G3P2		Sjogren's syndrome		ICP		Anti-SSA/Ro-52, ANA, PCNA
**Cases**	**Diagnosis means**	**GWs at diagnosis**	**AVI (ms)**	**Complications**	**CVPS**	**Therapy**	**Adverse effects**			
**Fetuses**										
Case 1-twin A	TDI Aa-Sa	25	170	EFE	Ten	Dex. + HCQ	Slight FGR^*^			
Case 1-twin B			160	EFE, hydrops	Ten → Nine					
Case 2	TDI Aa-Sa	19	163	No	Ten	Dex. + HCQ	Slight FGR			
Case 3	TDI Aa-Sa	21^+2^	172	No	Ten	Dex. + HCQ	Slight FGR			
Case 4	TDI Aa-Sa	28^+5^	170	No	Ten	Dex. + HCQ	Slight FGR			
**Cases**	**Delivery means**	**GWs at birth**	**Gender**	**Birth weight (g)**	**Birth length (cm)**	**Apgar scores**	**Autoantibody in serology**	**PR intervals on ECG (ms)**	**Neonatal therapy**	**Follow up**^**#**^ **(m)**
**Newborns**										
Case 1-twin A	Cesarean	35^+4^	Male	2,220	47	Ten	Anti-SSA/Ro	118	No	46
Case 1-twin B	Cesarean		Male	1,980	47	Ten	Anti-SSA/Ro	120	No	46
Case 2	Cesarean	37	Male	1,980	41	Ten	Anti-SSA/Ro and ANA	120	No	53
Case 3	Cesarean	38^+^	Female	2,350	49	Ten	Anti-SSA/Ro	110	No	41
Case 4	Cesarean	37^+1^	Female	2,510	47	Ten	Anti-SSA/Ro	124	No	8

*AVB, atrioventricular block; AVI, atrioventricular intervals; AHA, antihistone antibody; ANA, antinuclear antibodies; CTD, connective tissue disease; CVPS, cardiovascular profile score; Dex., dexamethasone; GWs, gestational weeks; HCQ, hydroxychloroquine; ICP, intrahepatic cholestasis of pregnancy; PCNA, proliferating cell nuclear antigen; SLE, systemic lupus erythematosus; TDI Aa-Sa, tissue Doppler imaging (TDI) from the onset of atrial contraction to ventricular systole (Aa-Sa)*.

* *FGR: fetal growth restriction (determined by measuring fetal biparietal diameters, femur lengths and head circumference, and abdominal circumference using ultrasound technique)*.

# *Follow up: the duration of postnatal follow-up (as the age of children until now, m, months), while all children had a normal development through catch-up growth*.

During gestation, comorbid diseases were only presented in case 4 with intrahepatic cholestasis of pregnancy. The relevant medicines for comorbid diseases were taken. Besides, the histories of other medicines or infection were denied, and amniotic fluid genetic screenings and virological tests such as TORCH were negative in all patients.

### Fetal Features

**Figure 1:**
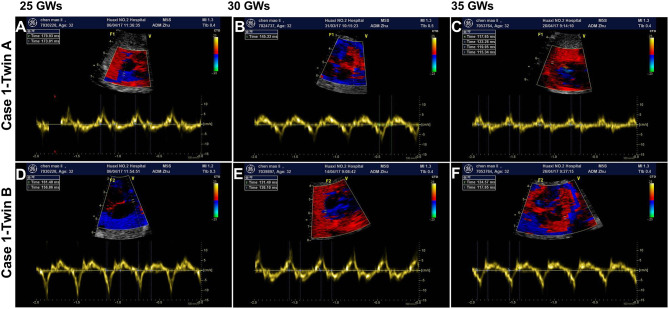
The findings of fetal atrioventricular interval monitored by fetal echocardiography. **(A,D)** Show the fetal atrioventricular (AV) intervals upon diagnosis (170/160 ms) at 25 gestational weeks (GWs). Panels B/E show the findings (145/136 ms) of medium term after treatment with oral dexamethasone at 30 GWs. **(C,F)** Show the final echocardiography prior to delivery (130/127 ms) at 35 GWs. **(A–C)** Belong to the fetus of case 1-twin A, and **(D–F)** belong to the fetus of case 1-twin B. ms, millisecond.

**Figure 2:**
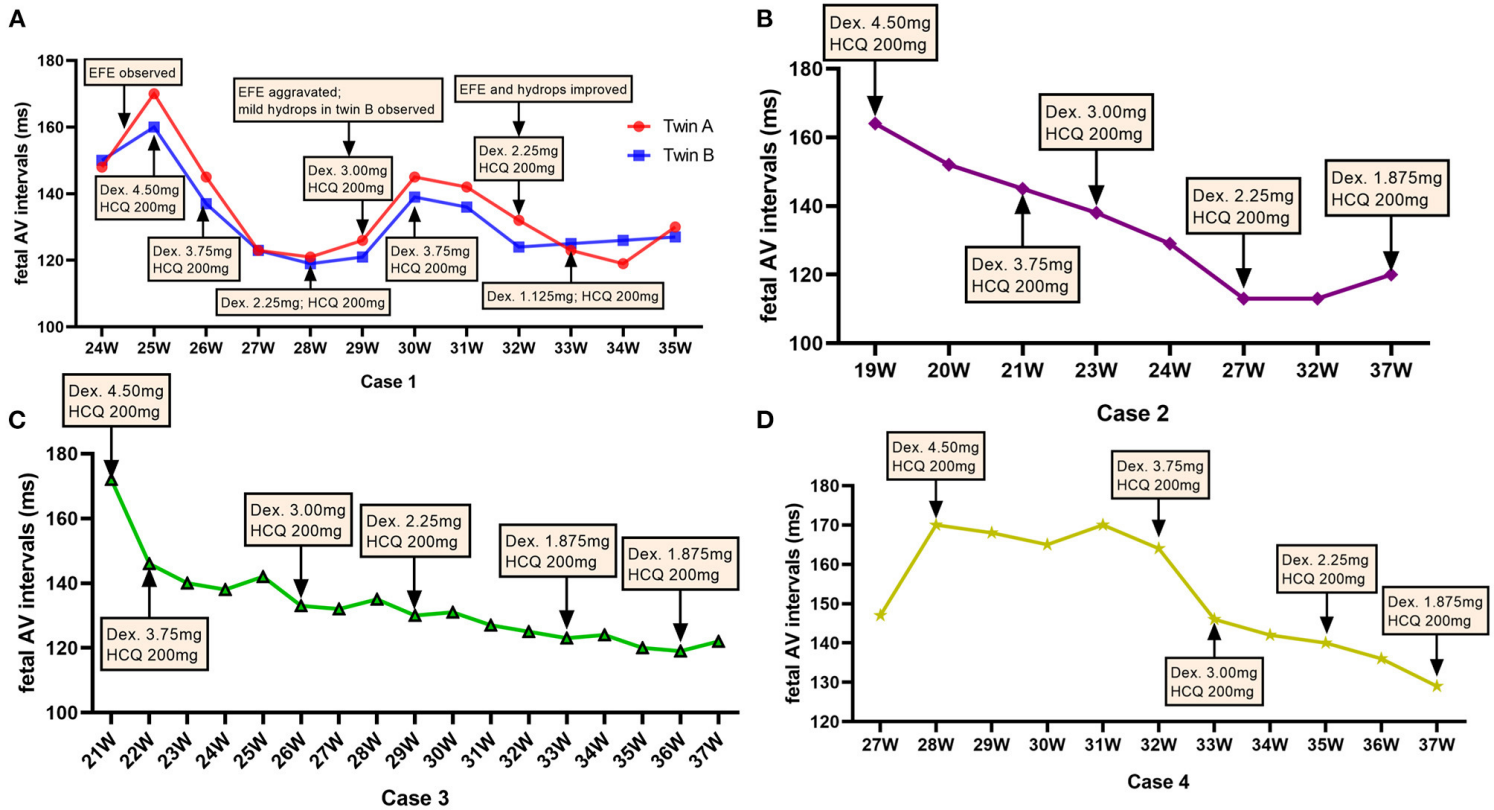
Dexamethasone dose adjustments and fetal atrioventricular intervals. The dose of dexamethasone (Dex) was adjusted according to the change in the fetal atrioventricular (AV) intervals at different gestational weeks (GWs) throughout the pregnancy. **(A)** Represents the fetuses of case 1; **(B)** represents the fetus of case 2; **(C)** shows the fetuses of case 3; **(D)** shows the fetus of case 4. mg, milligram.

The initial AV intervals in five fetuses were 170 ms (twin A, case 1), 160 ms (twin B, case 1), 163 ms (case 2), 172 ms (case 3), and 170 ms (case 4) at 25, 19, 21^+2^, and 28^+5^ gestational weeks (GWs), respectively. No structural cardiac disorders were found and the cardiovascular profile scores (CVPS) (13) in five fetus were 10. Additionally, the twin (case 1) suffered from EFE. Oral dexamethasone (4.5 mg daily as initial dose) and HCQ (200 mg bid) as well as weekly follow-up surveillance with fetal ultrasound (Figure 1 as the example of the twins) were performed until to the end of the pregnancy in our center. Dexamethasone dose was adjusted with the changes of fetal AV intervals and fetal development of biparietal diameters (BPD) and femur lengths (FL), as shown in Figure 2. Of concern, before admitting into our hospital, the mother in case 1 had received the intravenous immunoglobulin (IVIG) (400 mg/kg/d, twice) at 20GWs, while fetal prolonged AV intervals could not be recovered.

After the initiation of dexamethasone, the fetal AV intervals gradually declined to normal range in 1–2 weeks and the dosage of dexamethasone was slowly decreased. However, at 29–30 GWs, fetal EFE were thereafter aggravated and increased AV intervals were observed in twins as well as fetal mild hydrops and the CVPS reduced to nine scores in case 1-twin B. Therefore, the dose of dexamethasone was increased again to 3.75 mg daily for the first mother. Subsequently, the EFE and hydrops were improved and fetal AV intervals were controlled well in twins. Until delivery, the dose of dexamethasone was decreased to 1.125 mg per day in twins and to 1.875 mg per day in case 2, case 3 and case 4, and the AV intervals in all five fetuses were normal.

**Figure 3:**
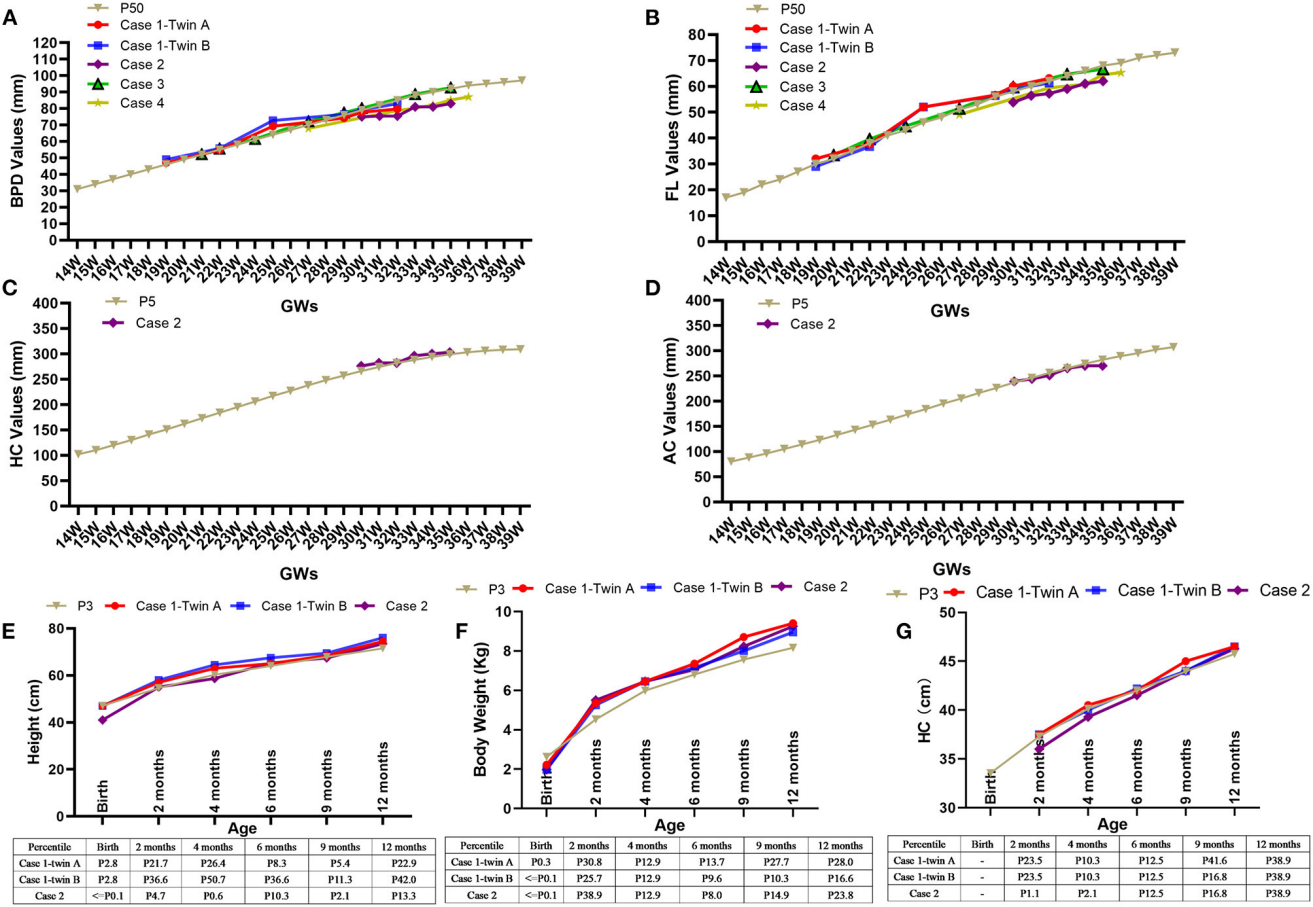
Adverse effects of dexamethasone on fetal growth and Catch-up growths during the first year of life. The fetal biparietal diameters (BPD) and femur lengths (FL) were ~50th percentile after the initiation of dexamethasone, as shown in five fetuses **(A,B)**. The changes in head circumference (HC) **(C)** and abdominal circumference (AC) **(D)** of the fetus in case 2 were ~5th percentile. The heights **(E)**, weights **(F)**, and head circumference (HC) **(G)** of the three infants in case 1 and case 2 during their monthly follow-up visits gradually increased and even exceeded the 3rd percentile *via* postnatally catch-up growth. GWs, gestational weeks; Kg, kilogram; cm, centimeter.

During the treatment, maternal and fetal adverse effects of dexamethasone were noted as diabetes in 1 mother and growth retardation in all fetuses. The QTc interval prolongation were not found in any of the pregnant women in our study. Fetal biparietal diameters (BPD), femur lengths (FL), head circumference (HC) and abdominal circumference (AC) were measured regularly at follow-up. We found the BPD and FL of five fetuses were ~50th percentile (Figures 3A,B), while the HC and AC of the fetus in case 2 were ~5th percentile (Figures 3C,D), revealing the growth retardation of these fetuses, especially the fetus in case 2.

### Newborn and Follow-Up

Five fetuses were delivered *via* cesarean section at 35^+4^, 37, 38, and 37^+1^ GWs due to premature rupture of membranes or placenta previa or abnormal position of fetus and low lying placenta. The birth weight was 2,220 g (Percentile [P] 0.3), 1,980 g (*P* ≤ 0.1), 1,980 g (*P* ≤ 0.1), 2,350 g (*P* 1.5), and 2,510 g (*P* 3.9) as well as the birth length was 47 cm (*P* 2.8), 47 cm (*P* 2.8), 41 cm (*P* ≤ 0.1), 49 cm (*P* 33.7), and 47 cm (*P* 5.5), respectively. [The percentiles were generated using the LMS method (14)]. Postnatally, instant electrocardiogram (ECG) was performed in five newborns with PR intervals of 118, 120, 120, 110, and 124 ms, respectively. Serological detection revealed positive anti-SSA antibodies rather than anti-SSB in all newborns. Additionally, there were no clinical or laboratory evidence of neonatal lupus syndrome such as hematological abnormalities, abnormal liver function and/or skin rash.

Monthly follow-up during the first year of life and afterwards twice a year were suggested. The serum anti-Ro/SSA antibody of all children could not be detected at the age of 4, 4, 5, 6, and 6 months, respectively. Measurements of height, body weight, and HC were used to estimate the effectiveness of postnatally catch-up growth, as shown in Figures 3E–G that the height and body weight gradually increased and exceeded 3rd percentile in case 1 and case 2. With a follow-up of 8–53 months, all children were healthy, with normal physical, mental and motor development, as well as normal echocardiography and ECG.

## Discussion

In this study, we firstly presented five fetuses diagnosed with first-degree AVB at 19–25 GWs in mothers with positive anti-SSA/Ro autoantibodies in China and treated using a combination therapy of dexamethasone with HCQ. These five fetuses had regularly monitoring since their diagnoses and were administered dexamethasone with doses adjusted according to fetal development and changes in the AV intervals. Fetal AV intervals were controlled well *in utero* and the postnatal outcomes were favorable. There was no progression and normal growth and development *via* catch-up growth was observed in all children. On the basis of the outcomes of our cases, it seems worth considering that dexamethasone combined with HCQ treat fetal first-degree AVB.

### Prenatal Diagnosis

Ultrasound techniques are now extensively validated and highly diagnostic of fetal arrhythmias and atrioventricular conduction abnormalities. Indeed, simultaneous pulse-wave Doppler (PD) interrogation of left ventricular inflow/outflow (in/out) or the superior vena cava/ascending aorta (V/AO) has been used to determine the functional integrity of the fetal AV conduction indirectly by their mechanical consequences (15, 16). However, the accuracy of measurements of flow-derived AV time intervals is influenced by loading condition, intrinsic myocardial properties, and the speed of pulse-wave propagation, as well as fetal position and orientation. As a consequence, this is often a challenging task, especially when there are limited acoustic windows or suboptimal fetal position. On the contrary, TDI is less load dependent, could provide better temporal resolution and allow more accurate analysis of segmental wall motion in any area of the heart during the same cardiac cycle. Moreover, previous studies have proved that TDI-derived intervals tracked the fetal AV intervals more closely than the other Doppler techniques and may be a more accurate ultrasound method for assessing fetal AV conduction (11, 16). TDI-derived AV interval could be measured from the atrial contraction (Aa) to isovolumetric contraction (IV) or to ventricular systole (Sa) at the base of the right ventricular wall (11). However, in clinical work, we found that it is relatively challenging to obtain a clear waveform of isovolumetric contraction and identifying the onset of isovolumetric contraction is difficult and always inaccurate. The reproducibility of Aa-Sa measurement was is much better than that of Aa-IV. Therefore, TDI-derived Aa-Sa was applied to determine fetal AV intervals and identify fetal first-degree AVB in our center.

The definitions of fetal first-degree AVB vary in previously different studies and the reference data of fetal AV intervals varies significantly in different approaches. On the basis of the normal values of TDI-derived Aa-Sa intervals in our previous study (in Chinese) (10) as described in the methods section, the definition of abnormal fetal AV interval was set a priori at 3SD (>150 ms) above the normal mean in our group. The AV intervals of five fetuses, included in our study, were over 160 ms, with three of them over 170 ms. A more stringent criterion for defining abnormal was used because we envisioned this threshold to represent a clinical trigger for labeling the fetus with a serious disease and potentially treating the maternal-fetal dyad with a drug associated with serious toxicity (dexamethasone). Additionally, a previous study (11) has found that the AV time measurement by Aa-Sa was longer than that of fetal PR interval since the ventricular pre-ejection period (time delay from Q wave to ventricular ejection) is longer than the atrial pre-ejection period (time delay from *P* wave to atrial ejection). Moreover, the study conducted by Sonesson et al. (12) had found that fetal AV intervals in the range of 135–140 ms spontaneously reverted and were not associated with clinical pathology. Therefore, we proposed that applying a lower cut-off value would lead to over-diagnosis and over-treatment of many fetuses. Collectively, the normal values of fetal AV intervals vary among different methods of measurement and could be affected by gestational weeks and fetal heart rate. Therefore, the most proper cut-off values for the diagnosis of fetal first-degree AVB remains to be defined in further studies.

### Prenatal Treatment

Fluorinated steroids, which are the initial choice to treat AVB, are a group of anti-inflammatory drugs that can cross the placenta and are available to the fetus in the active form (17, 18). However, to our knowledge, there were no consensus in terms of the choice of steroids types (dexamethasone or betamethasone), initial drug dosage, the criteria used to adjust steroid dosage and the choice of the subsequent dose to administer. Dexamethasone was used in most of previous studies and also adopted in our group. For case 1 in our study, after the initiation of dexamethasone (4.5 mg per day) therapy at 25 GWs, the fetal AV intervals of both twins declined to normal range 1 week later and the dosage of dexamethasone was decreased to 3.75 mg per day at 26 GWs. Thereafter, we rapidly reduced the dosage of dexamethasone from 3.75 to 2.25 mg in 2 weeks. Unfortunately, it was found that the fetal AV intervals of the twins increased again at 29 GWs. Moreover, fetal EFE were aggravated in both twins and fetal hydrops was observed in twin-B. We speculated that the recurrence of the disease might be partly attributable to the rapid dosage reduction of dexamethasone. On the basis of these experiences, for subsequent included cases, slower dosage reduction of dexamethasone (0.75 mg each time every 2–4 weeks) was applied if the fetal AV interval were stable. But, obvious signs of fetal growth restriction and decreasing volume of amniotic fluid were considered indications to decrease the dosage at a faster rate. For instance, for case 4, after usage of dexamethasone with a dosage of 4.5 mg per day for 4 weeks (from 28 to 32 GWs), fetal AV interval began to gradually decline and the dosage was reduced to 3.75 mg per day at 32 GWs. However, obvious fetal growth restriction was observed and the dosage was rapidly decreased to 3.0 mg in 1 week. Thereby, we considered that the dosage adjustment regimen of dexamethasone should be individualized mainly according to the fetal AV intervals, cardiac function as well as the side effects of dexamethasone (19). Despite the anti-Ro/SSA was detectable in all newborns, there were no clinical or laboratory evidence of neonatal lupus syndrome. Considering the potential side-effects of steroids for neonates, postnatal dexamethasone therapy for newborns with positive anti-Ro/SSA was not adopted in our center and only monthly follow-up was suggested during the first year after delivery.

Except for steroids, HCQ was significantly associated with a decreased risk of AVB in fetuses of mothers with autoimmune disorders and may be transported across the placenta to directly modulate autoimmune-mediated inflammation in fetal hearts (20–22). Therefore, in our group, HCQ was suggested for the pregnant women once the autoimmune disease was diagnosed and continued during the remainder of the pregnancy. In terms of IVIG usage, the mother in case 1 had received IVIG infusion (400 mg/kd/d, twice) at 20 GWs, while fetal prolonged AV intervals could not be recovered. In our center, IVIG infusion was not adopted since there is currently no evidence for IVIG therapy in autoimmune associated first-degree AVB so far.

Several limitations should be addressed. Owing to the lack of control group (untreated group) and limited sample size, it was difficult to prove progression of higher-degree AVB could be prevented by fluorinated steroids and HCQ in our study. As summarized in our recent review (23), fetal autoimmune-associated first-degree AVB could revert to normal or maintained unchanged without treatment. Indeed, the twin in our study presented with no progression without therapy (as IVIG is not a proven therapy). However, advanced block has been documented within days of a previously normal fetal echocardiogram and weekly surveillance is not sufficiently frequent to identify a proper therapeutic window (24). More importantly, the cases of first-degree AVB directly developed to third-degree AVB had been reported (12, 25). Therefore, given the adverse outcome of third-degree AVB and the relatively favorable results of our study, prenatal treatment for fetal autoimmune-associated first-degree AVB could be worthy of consideration. However, strict surveillance and timely adjustment of the treatment according to the conditions of the mother and the fetus are indicated. Further studies are necessary to prove our concept.

### Conclusion

Nowadays, the definition of fetal first-degree AVB remains to be established with proper technique and the necessity of the treatment *in utero* for fetal autoimmune-associated first-degree AVB is still controversial (16). In this study, we shared our experiences that the AV intervals in five fetuses diagnosed with autoimmune-associated first-degree AVB were normalized through the combination therapy of dexamethasone with HCQ. Based on the weekly follow-up by fetal echocardiography, the dose adjustment controlled the fetal AV intervals. Maternal and fetal adverse effects of dexamethasone were noted as diabetes in 1 mother and growth retardation in the fetuses, however, more severe adverse effects could perhaps be limited by our dose adjustment regime. Prenatal treatment for fetal autoimmune-associated first-degree AVB could be an alternative under the strict surveillance and timely adjustment of the treatment according to the conditions of the mother and the fetus. However, the most proper diagnostic and therapeutic management warrant to be further proven in a randomized trial.

## Data Availability Statement

The original contributions generated for the study are included in the article/supplementary material, further inquiries can be directed to the corresponding authors.

## Ethics Statement

The studies involving human participants were reviewed and approved by the University Ethics Committee on Human Subjects at Sichuan University (2010002). The patients/participants provided their written informed consent to participate in this study. Written informed consent was obtained from the individual(s), and minor(s)' legal guardian/next of kin, for the publication of any potentially identifiable images or data included in this article.

## Author Contributions

CW, YH, KZ, and YL: conceptualization. CT, HY, QZ, and MC: resources. CT, CW, and SS: formal analysis and investigation. CW, YH, and KZ: funding acquisition. CT and HY: writing-original draft preparation. KZ and CW: writing-review and editing. All authors contributed to the article and approved the submitted version.

## Conflict of Interest

The authors declare that the research was conducted in the absence of any commercial or financial relationships that could be construed as a potential conflict of interest.
